# Psychological Well-Being and Mental Health in Youth: Technical Adequacy of the Comprehensive Inventory of Thriving

**DOI:** 10.3390/children10071269

**Published:** 2023-07-23

**Authors:** Gökmen Arslan

**Affiliations:** 1 Department of Psychological Counseling, Mehmet Akif Ersoy University, Burdur 15100, Türkiye; gkmnarslan@gmail.com; 2 Centre for Wellbeing Science, University of Melbourne, Melbourne, VIC 3010, Australia

**Keywords:** psychological well-being, mental health, thriving, youths, wellbeing

## Abstract

Well-being is a crucial construct in young people’s lives that directly affects their overall quality of life, academic performance, and social relationships. Although there is an emphasis on the significance of positive psychological states in mental health, few have focused on positive states, and psychological well-being is often conceptualized using negative indicators of mental health. The present study aims to fill the gap in the literature by testing the reliability and validity of the Turkish version of the Comprehensive Inventory of Thriving for Youth (CIT-Y) and exploring the relationship between psychological well-being and mental health problems among Turkish young people. The study involved 459 youths from a state elementary school in a city in Türkiye, with 48.8% (224 females and 235 males) of them being female and aged between 11 and 15 years (*M* = 12.85, *SD* = 0.73). Findings from the study suggest that the CIT-Y is a reliable and valid measure for assessing psychological well-being in Turkish young people. Additionally, the results show that young people with internalizing and externalizing problems report fewer positive psychological states compared to those without such problems. This emphasizes the significance of well-being domains, including loneliness and respect, in comprehending mental health issues among young people. These findings can aid mental health providers in designing interventions to enhance the psychosocial adjustment of students by providing resources to cultivate mental health and well-being.

## 1. Introduction

In recent years, there has been a rising interest in the psychological well-being of young people among researchers and practitioners, both in theory and in practice. While early interventions for mental health disorders are crucial for reducing their ongoing effects and long-term consequences [1,2], it has been emphasized that mental health is not simply the absence of psychological symptoms but also encompasses the presence of positive psychological states [3,4]. Although there is an emphasis on the significance of positive psychological states in mental health, some scholars have pointed out that the majority of research has primarily evaluated psychological well-being using negative indicators of mental health, such as depressive symptoms, anxiety, or stress [5,6,7,8,9]. Therefore, it is essential to evaluate psychological well-being, as it is integral to the creation of significant policies that focus on enhancing the quality of life for children and adolescents [10,11,12]. Additionally, considering the impacts of adverse experiences, such as the COVID-19 pandemic, on young people’s mental health and well-being [13,14], it is crucial for mental health professionals to recognize these effects and develop targeted interventions to support the psychological well-being of young people during and after such challenging experiences [15]. The evaluation of youth well-being enables researchers to identify the factors within their home, school, environment, and society that foster their growth and success. Furthermore, creating more suitable assessment tools that enable young people to express their well-being would enable the measurement of the overall quality of early childhood programs using broader metrics [10]. The present study aimed to address this need by examining the psychometric properties of the Comprehensive Inventory of Thriving (CIT) [16] with Turkish youths. The study also intended to investigate the association between positive and negative psychological states and mental health problems among young people.

### Psychological Well-Being in Youths

Well-being is a crucial construct in people’s lives and is widely characterized by two major aspects: hedonic and eudaimonic well-being. Hedonic well-being, often referred to as subjective well-being, refers to the feeling of pleasure and enjoyment that is derived from experiences, while eudaimonic well-being, often referred to as psychological well-being, is based on the idea that individuals experience happiness when they have a sense of purpose in life, encounter challenges, and undergo personal growth [17,18]. Subjective well-being is conceptualized as the subjective experience of feeling satisfied, having a prevalence of positive feelings, and having a scarcity of negative feelings [19,20]. In contrast to hedonic well-being, there is no single approach to studying eudaimonic well-being [17]. However, all approaches focus on factors related to growth and meaning while excluding those related to affect [18]. For example, Ryff and Keyes [21] have conceptualized psychological well-being based on six aspects of wellbeing: environmental mastery, autonomy, personal growth, purpose in life, positive relations with others, and self-acceptance. Further, Seligman [22] suggested the PERMA model as a simplified version of the broader concept of well-being, including both hedonic and eudaimonic aspects. It consists of five components: positive emotions (P), engagement (E), relationships (R), meaning (M), and accomplishment (A). Butler and Kern [23] developed a tool to evaluate overall well-being that builds on Seligman’s PERMA model. More recently, Su et al. [16] conducted a comprehensive review of the literature on psychological well-being and outlined seven key dimensions associated with thriving. These dimensions include subjective well-being encompassing life satisfaction, negative and positive emotions, active engagement in daily activities, finding meaning and purpose in life, fostering supportive positive relationships involving social support, respect, community, belongingness, trust, and addressing loneliness, maintaining autonomy, cultivating a sense of mastery and accomplishment through skills, self-efficacy, learning, and self-worth, and fostering optimism. The term “thriving” is defined as “the state of positive functioning at its fullest range—mentally, physically, and socially” [12] (p. 256) and reflects the concept of psychological well-being. Consequently, the Comprehensive Inventory of Thriving (CIT) was developed to evaluate various constructs related to well-being [16,24]. The 18 latent constructs were measured by utilizing sub-scales of thriving, each comprising three items. By integrating key aspects of both hedonic and eudaimonic approaches, the measure provides a comprehensive framework for understanding positive psychological functioning and well-being [24].

Existing literature has indicated that there are some assessment tools available to measure well-being in children and adolescents [25,26,27]. However, these tools are typically designed for specific purposes and focus only on a limited number of aspects, such as positive emotions or life satisfaction, and are primarily developed to assess well-being in adolescents and adults (see, [26,28]). Therefore, it is essential to validate such inventories of psychological well-being to develop a better understanding of how to promote youth well-being and mental health globally. Su et al. [16] have highlighted the potential of the CIT as a screening tool to identify individuals with varying levels of psychological well-being. Despite the comprehensive framework it provides, the original structure of 18 factors has not been widely replicated, especially in children and adolescents. Previous studies have provided evidence suggesting that the CIT is a reliable and valid measure for assessing psychological well-being in young adults and adults from different cultures [8,24,29,30,31]. However, very few studies have focused on its psychometrics for children and adolescents. To the best of our knowledge, only one study has examined the validity and reliability of the CIT with children. The study indicated that the measure was psychometrically reliable and valid for assessing psychological well-being among Italian children [10]. The present study aims to fill the gap in the literature by testing the reliability and validity of the Turkish version of the Comprehensive Inventory of Thriving for Youth (CIT-Y). Additionally, the study aims to explore the relationship between psychological well-being and mental health problems and investigate the positive and negative psychological states across mental health statuses among young people.

## 2. Method

### 2.1. Participants

This cross-sectional study, utilizing a convenience sampling method, involved 469 youths from a public elementary school in a city in Türkiye. After excluding missing data and poorly completed surveys, the sample included 259 children, with 48.8% (224 females and 235 males) of them being female and aged between 11 and 15 years (*M* = 12.85, *SD* = 0.73).

### 2.2. Procedures

The participants were provided with a paper-and-pencil survey comprising the study questionnaires and demographic items. The survey was administered to students who volunteered to participate in the study. Prior to data collection, all participants underwent a review of the informed consent form, indicating their understanding and agreement. The study received approval from the institutional review board. Subsequently, the dataset was screened, and poorly completed surveys as well as instances of missing data (missing data > 10%) were removed.

### 2.3. Measures

*The Comprehensive Inventory of Thriving for Youth (CIT-Y).* The CIT is a self-reported measure used to assess psychological well-being [16]. The CIT has 54 items and 18 subscales, each consisting of three items that map onto seven psychological constructs that represent the higher-order latent construct of thriving. The items are rated on a 5-point scale ranging from strongly disagreeing (1) to strongly agreeing (5), with higher scores indicating greater psychological wellbeing. The brief version of the CIT, the Brief Inventory of Thriving (BIT), has 10 items that reflect the same psychological well-being domains. Previous research has shown that both versions of the inventory have acceptable data-model fit statistics and internal reliability estimates in different cultures [8,16,24]. Concurrent and predictive validity have also been supported by previous research [8,16].

While the inventory has been translated into Turkish for young adults [29], there is no evidence of its psychometric properties with Turkish children and adolescents. For this study, the Turkish version of the CIT created by Arslan (2021) was used. However, before administering the measure, two independent experts in school and counseling psychology reviewed the translation to ensure developmental and readability considerations were taken into account. Based on their comments, two items were slightly revised and updated in the form. The final Turkish version of the CIT for youth included 54 items rated on a 5-point scale, consistent with the original version.

*Strengths and Difficulties Questionnaire (SDQ).* The SDQ is a tool that was created to evaluate emotional and behavioral issues, as well as wellbeing indicators, in young people [32]. It consists of 25 self-reported items and includes five subscales that measure emotional problems, conduct problems, peer problems, hyperactivity, and prosocial behavior. The items are rated using a 3-point scale, with 0 indicating “not true”, 1 indicating “somewhat true”, and 2 indicating “certainly true”. Güvenir et al. [33] conducted a study on the SDQ’s validity and reliability with Turkish youth and found that the internal consistency coefficients of the scales ranged between 0.65 and 0.84, except for the peer problems factor, which had a lower internal consistency (α 0.37) than the other scales. The categorization of mental health status groups was also determined by utilizing the cut-off score of the SDQ for internalizing and externalizing problems [32].

### 2.4. Data Analyses

Initially, confirmatory factor analysis was utilized to validate the factor structure of both the CIT and BIT as defined in the developmental study of the inventories [16]. Various data-model fit statistics, including the root mean square error of approximation (RMSEA) and the standardized root mean squared residual (SRMR) ≤ 0.08 and the comparative fit index (CFI) ≥ 0.90, were used to assess the measurement model’s goodness of fit [34,35], indicating adequate data-model fit. After confirming the measures’ factor structure, descriptive statistics and the correlations between thriving constructs and youth mental health indicators were examined. The skewness and kurtosis scores and their cutoff values were used to examine the normality assumption for the study’s measures [36,37]. Network analysis was also conducted to visually depict the intercorrelations among the study variables using JASP 0.17.10 [38]. Finally, young people were divided into two groups using the cutoff scores of the SDQ to compare the groups’ effects. A series of independent t-test analyses was conducted to compare psychological well-being constructs in “*at-risk*” and “*typical*” groups for internalizing and externalizing problems. were used to compare the two groups based on psychological well-being constructs. The effect sizes for Cohen’s *d* were assessed using the decision points: small d =  0.2, medium d  =  0.5, and large d  =  0.8 [39]. All statistical analyses were performed using AMOS v24 and IBM SPSS Statistics v27.

## 3. Results

A confirmatory factor analysis was conducted to examine the factor structure of the CIT. The CIT was comprised of 54 items that measured 18 constructs related to thriving, such as support, learning, and life satisfaction. Results indicated that the model fit was adequate based on several data-model fit statistics—*χ*^2^ = 2174.74, *df* = 1224, *p* < 0.001, CFI = 0.91, RMSEA [95% CI] = 0.041 [0.038, 0.044], SRMR = 0.045. Additionally, factor loadings for the CIT items ranged between 0.33 and 0.92, indicating adequate-to-strong loadings, as seen in Table 1. Moreover, the factor analysis of the brief version of the measure—the Brief Inventory of Thriving (BIT)—showed that the model fit was acceptable—*χ*^2^ = 158.08, *df* = 35, *p* < 0.001, CFI = 0.90, RMSEA [95% CI] = 0.087 [0.074, 0.10], SRMR = 0.054. Factor loadings for the BIT items were found to be adequate-to-good, ranging from 0.37 to 0.66.

**Figure 1:**
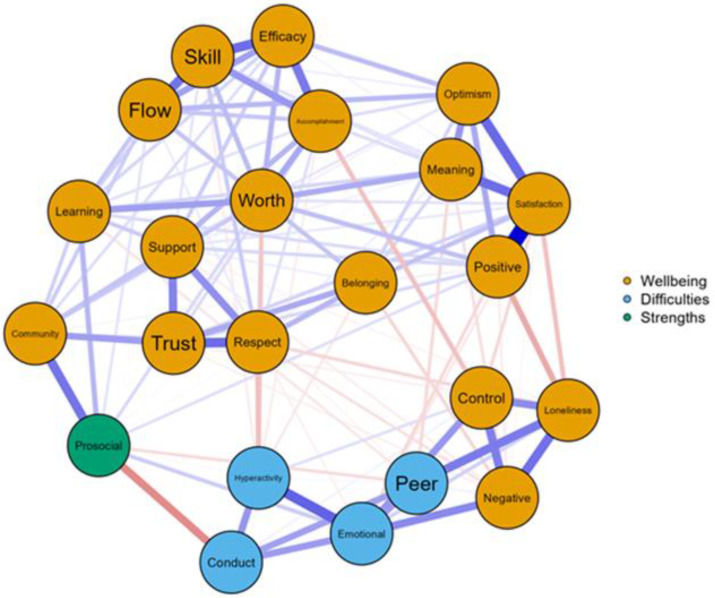
EBICglasso network analysis.

After establishing the factor structure of the CIT and BIT, we examined descriptive statistics, normality assumptions, and internal reliability estimates of the inventories. The skewness and kurtosis values suggested a relatively normal distribution of all variables, with internal reliability estimates ranging from adequate to strong, as shown in Table 2. Network analysis was further conducted to visually depict the associations among the study variables. Findings from the network analysis showed that the nodes with community and prosocial behaviors had the strongest edge intensity (*r* = 0.20). Hyperactivity had the strongest negative association with respect (edge weight = −0.09). With regard to emotional problems, negative feelings (edge weight = 0.17) and lack of control (edge weight = 0.15) were the most central nodes. Peer problems were strongly associated with loneliness (edge weight = 0.19), as illustrated in Figure 1. The correlation analysis results also provided evidence supporting the concurrent validity of the inventories among young Turkish people, as presented in Table 3.

In the final step, a set of independent t-test analyses was used to compare the impact of mental health groups on young people’s psychological well-being constructs, as presented in Table 4 and Table 5. The results of these analyses demonstrated a significant overall effect of mental health levels (i.e., internalizing and externalizing problems) on all constructs, with small to large Cohen’s *d* effect sizes. With regards to externalizing problems, Cohen’s *d* effect sizes ranged from 0.25 for meaning to 0.62 for negative feelings. Regarding internalizing problems, the effect sizes were between 0.30 for belonging and 0.85 for loneliness. Furthermore, the findings revealed a significant effect of mental health levels on the BIT, with a *d* effect size of 0.52 for externalizing and 0.72 for internalizing scores. These results suggest that at-risk young people with internalizing and externalizing symptoms exhibit lower levels of positive psychological well-being constructs and greater negative domains of psychological well-being than those with typical symptom levels.

## 4. Discussion

Psychological well-being is crucial for young people as it directly affects their overall quality of life, academic performance, and social relationships. Although there is an emphasis on the significance of positive psychological states in mental health, few have focused on positive states, and psychological well-being is often conceptualized using negative indicators of mental health [7,8]. Therefore, it is a critical step to measure psychological well-being, as it is integral to creating significant policies that focus on enhancing mental health for children and adolescents [10,11,12]. Measuring psychological well-being in youths can contribute to developing strategies that help promote positive mental health and prevent the development of mental health issues. Further, given the significant influence of culture on how people develop, manifest, identify, and express their feelings, thoughts, and behaviors [2,40], developing and adapting a measure for use in a specific culture can reduce cultural bias and increase the validity and reliability of the measure. The present study aimed to examine the reliability and validity of the Turkish version of the Comprehensive Inventory of Thriving for Youth (CIT-Y) and to explore the relationship between psychological well-being and mental health problems.

Factor analysis first revealed that the CIT had psychometrically good data-model fit statistics, confirming the latent structure of the inventory. Factor loadings for the CIT items ranged between 0.33 and 0.92, indicating adequate-to-strong loadings. Moreover, the measure had adequate-to-strong internal reliability estimates. Consistent with the results of this study, some research found that the CIT provided adequate psychometric properties in different cultures, such as Brazilian [31], German [8], Turkish [8,29], Chinese [30], and Italian [24] adult samples. However, very few have examined the validity and reliability of the measure for children and adolescents. While the original study by Su et al. [16] and the current study confirmed the 18-factor structure of the CIT, Andolfi et al. [10] found an alternative 12-factor structure of the CIT specifically for children. Community, trust, and self-efficacy were excluded from the measure because of low internal reliability estimates in Italian children. Consistent with the findings of this study, internal reliability estimates of the Italian version of the CIT for children also ranged from adequate to strong [10]. Collectively, the results of this study provide reliability and validity evidence that support the CIT as an effective and robust tool for assessing psychological well-being among Turkish young people.

Regarding the relationship between psychological well-being and mental health problems, both internalizing and externalizing problems were found to have significant and positive correlations with negative domains of psychological well-being such as loneliness and negative feelings and negative correlations with positive domains of well-being such as belonging, support, life satisfaction, and self-efficacy. The network analysis also revealed that prosocial behavior was the most central node for community, while hyperactivity had the strongest negative association with respect. Negative feelings and a lack of control were identified as the most central nodes for internalizing problems, and peer problems were strongly associated with loneliness. Furthermore, young people who were at risk for internalizing and externalizing problems reported relatively greater negative psychological well-being states and fewer positive well-being states than those in the typical group. Youths with mental health problems are more likely to experience less psychological well-being than those without. The results of this study align with the complete mental health approach [3,4], highlighting the importance of considering positive psychological states in addition to the absence of psychological symptoms when assessing mental health. Andolfi et al. [10] also reported significant associations between well-being measures and children’s life satisfaction in domains such as family, friends, and affective well-being. Furthermore, young adults with severe psychological symptoms reported lower levels of positive psychological well-being and higher levels of negative well-being domains compared to those with mild symptoms [29]. Su et al. [16] found an association between psychological well-being and various mental, physical, and behavioral problems. Individuals with higher well-being scores experienced better health status, fewer medical problems, higher physical functioning, and engaged in more frequent health behaviors. Overall, these findings underscore the need for a comprehensive approach to mental health that considers both the presence of positive states and the absence of negative symptoms.

## 5. Implications and Limitations

The present study suggests that both the CIT and the BIT are reliable and valid measures for assessing psychological well-being among Turkish young people. Furthermore, the findings indicate that youths with internalizing and externalizing problems have fewer positive psychological states than those without such problems. The study highlights the importance of well-being domains such as loneliness and respect in understanding mental health problems among young people. Evaluations of psychological well-being provide a valuable tool to create suitable measures for preventing and intervening in school settings, with the aim of enhancing the positive academic and psychosocial experiences of young people. School-based mental health providers could utilize the inventories to provide comprehensive screenings of psychological well-being in children. The utilization of the complete mental health approach [11,41] suggests that combining inventories measuring negative psychological states with those measuring positive states can offer a more thorough understanding of psychological well-being. Results from the study indicate that young people with internalizing and externalizing issues have fewer positive psychological states and more negative ones. These findings can aid mental health professionals in designing interventions to enhance the psychosocial adjustment of students by providing resources to promote youth mental health and well-being. Therefore, this study provides further evidence for the use of both the CIT and BIT to assess the fundamental elements of psychological well-being, which can be valuable for researchers and practitioners working to promote youth mental health and well-being in school settings.

Although the present study has important implications for both research and practice, there are a few limitations that need to be considered. Firstly, the study utilized a cross-sectional approach, which does not allow for causal inferences. Thus, future research should employ longitudinal research methods to investigate the variables associated with psychological well-being. Secondly, the sample size of young people was derived from a convenience sample at a state elementary school in Türkiye, which limits the generalizability of the findings to other contexts. Therefore, further studies are needed to examine the thriving model in diverse populations and could design cross-cultural research to gain a deep understanding of psychological well-being in youths. Another limitation of this study is that data collection was carried out using self-reported instruments. Self-reported measures rely on participants’ subjective perceptions and may be influenced by factors such as social desirability bias or recall bias. Therefore, the findings should be interpreted with caution, considering the potential limitations associated with self-reported data. Finally, in addition to student internalizing and externalizing symptoms, different indicators of mental health and quality of life (e.g., academic success, substance misuse) could be used to provide additional validity evidence and to better understand the relationships between psychological well-being domains and outcomes.

## Figures and Tables

**Table 1:** Confirmatory factor analysis results.

Factor	Indicator	Stand. Estimate
Support	There are people I can depend on to help me	0.65
	There are people who give me support and encouragement	0.80
	There are people who appreciate me as a person	0.71
Community	I pitch in to help when my local community needs…	0.70
	I invite my neighbors to my home	0.59
	I look for ways to help my neighbors when they are in need	0.65
Trust	I can trust people in my society	0.57
	People in my neighborhood can be trusted	0.65
	Most people I meet are honest	0.77
Respect	People respect me	0.63
	People are polite to me	0.82
	I am treated with the same amount of respect as others	0.75
Loneliness	I feel lonely	0.57
	I often feel left out	0.81
	There is no one I feel close to	0.78
Belonging	I feel a sense of belonging in my community	0.50
	I feel a sense of belonging in my state or province	0.73
	I feel a sense of belonging in my country	0.78
Engagement	I become fully absorbed in activities I do	0.66
	In most activities I do, I feel energized	0.71
	I become excited when I work on something	0.60
Skills	I use my skills a lot in my everyday life	0.52
	I frequently use my talents	0.80
	I get to do what I am good at everyday	0.76
Learning	I learned something new yesterday	0.66
	Learning new things is important to me	0.61
	I always learn something everyday	0.60
Lack of control	Other people decide most of my life decisions (R)	0.61
	The life choices I make are not really mine (R)	0.77
	Other people decide what I can and cannot do (R)	0.79
Accomplishment	I am achieving most of my goals	0.71
	I am fulfilling my ambitions	0.74
	I am on track to reach my dreams	0.72
Self-Efficacy	I can succeed if I put my mind to it	0.70
	I am confident that I can deal with unexpected events	0.52
	I believe that I am capable in most things	0.34
Self-Worth	What I do in life is valuable and worthwhile	0.65
	The things I do contribute to society	0.76
	The work I do is important for other people	0.67
Meaning	My life has a clear sense of purpose	0.66
	I have found a satisfactory meaning in life	0.69
	I know what gives meaning to my life	0.59
Optimism	I am optimistic about my future	0.74
	I have a positive outlook on life	0.77
	I expect more good things in my life than bad	0.46
Life satisfaction	In most ways my life is close to my ideal	0.52
	I am satisfied with my life	0.33
	My life is going well	0.78
Positive feelings	I feel positive most of the time	0.81
	I feel happy most of the time	0.90
	I feel good most of the time	0.85
Negative feelings	I feel negative most of the time (R)	0.73
	I experience unhappy feelings most of the time (R)	0.92
	I feel bad most of the time (R)	0.92

**Table 2:** Descriptive statistics of the measures.

	*Mean*	*SD*	Skewness	Kurtosis	α	ω
CIT	–	–	–	–	0.93	0.93
Support	11.64	2.66	−0.83	0.55	0.77	0.76
Community	11.41	2.56	−0.73	0.36	0.69	0.68
Trust	8.79	2.59	−0.14	−0.05	0.71	0.70
Respect	10.08	2.64	−0.28	0.08	0.77	0.77
Loneliness	7.78	3.16	0.23	−0.62	0.77	0.76
Belonging	11.38	2.63	−0.60	−0.04	0.73	0.71
Flow	10.96	2.39	−0.18	−0.24	0.70	0.70
Skills	10.13	2.48	0.06	−0.28	0.73	0.71
Learning	11.13	2.75	−0.53	0.29	0.66	0.65
Lack of control	7.05	3.15	0.50	−0.38	0.84	0.84
Accomplishment	10.39	2.61	−0.38	0.02	0.77	0.77
Self-efficacy	10.92	2.38	−0.54	0.57	0.73	0.72
Self-worth	9.88	2.44	−0.10	0.33	0.73	0.71
Meaning	10.25	2.68	−0.18	−0.20	0.73	0.73
Optimism	10.82	2.63	−0.57	0.48	0.71	0.69
Life satisfaction	10.31	2.74	−0.38	−0.02	0.76	0.74
Positive feelings	10.32	2.98	−0.47	−0.04	0.89	0.88
Negative feelings	8.74	3.24	−0.01	−0.57	0.89	0.89
BIT	35.66	6.75	−0.29	0.16	0.82	0.82

**Table 3:** Correlation results.

	1	2	3	4	5	6	7	8	9	10	11	12	13	14	15	16	17	18	19	20	21	22
1. Support	–	0.39 **	0.50 **	0.50 **	−0.33 **	0.34 **	0.35 **	0.42 **	0.39 **	−0.27 **	0.48 **	0.44 **	0.44 **	0.39 **	0.42 **	0.46 **	0.44 **	−0.31 **	0.63 **	−0.19 **	−0.26 **	0.25 **
2. Community		–	0.40 **	0.37 **	−0.16 **	0.27 **	0.34 **	0.33 **	0.34 **	−0.11 *	0.34 **	0.35 **	0.38 **	0.21 **	0.33 **	0.31 **	0.29 **	−0.13 **	0.42 **	−0.18 **	−0.15 **	0.37 **
3. Trust			–	0.51	−0.32 **	0.39 **	0.24 **	0.34 **	0.25 **	−0.18 **	0.40 **	0.33 **	0.38 **	0.29 **	0.36 **	0.45 **	0.42 **	−0.30 **	0.50 **	−0.21 **	−0.26 **	0.16 **
4. Respect				–	−0.35 **	0.39 **	0.35 **	0.42 **	0.26 **	−0.26 **	0.43 **	0.39 **	0.41 **	0.37 **	0.37 **	0.43 **	0.35 **	−0.33 **	0.55 **	−0.30 **	−0.31 **	0.22 **
5. Loneliness					–	−0.22 **	−0.16 **	−0.25 **	−0.27 **	0.43 **	−0.30 **	−0.22 **	−0.29 **	−0.29 **	−0.27 **	−0.46 **	−0.45 **	0.48 **	−0.45 **	0.31 **	0.47 **	−0.14 **
6. Belonging						–	0.33 **	0.28 **	0.26 **	−0.15 **	0.30 **	0.23 **	0.30 **	0.35 **	0.37 **	0.42 **	0.36 **	−0.29 **	0.54 **	−0.21 **	−0.18 **	0.19 **
7. Engagement							–	0.51 **	0.37 **	−0.12 *	0.45 **	0.48 **	0.38 **	0.33 **	0.42 **	0.34 **	0.35 **	−0.12 *	0.59 **	−0.16 **	−0.14 **	0.25 **
8. Skills								–	0.40 **	−0.18 **	0.54 **	0.56 **	0.49 **	0.40 **	0.43 **	0.45 **	0.38 **	−0.19 **	0.59 **	−0.22 **	−0.26	0.19 **
9. Learning									–	−0.12 *	0.40 **	0.40 **	0.45 **	0.36 **	0.35 **	0.37 **	0.38 **	−0.24 **	0.50 **	−0.24 **	−0.20 **	0.31 **
10. Lack of cont.										–	−0.32 **	−0.20 **	−0.18 **	−0.15 *	−0.17 **	−0.36 **	−0.27 **	0.44 **	−0.35 **	0.32 **	0.41 **	−0.12 *
11. Accomplis.											–	0.56 **	0.51 **	0.42 **	0.46 **	0.47 **	0.41 **	−0.23 **	0.72 **	−0.26 **	−0.26 **	0.25 **
12. Self-efficacy												–	0.49 **	0.29 **	0.46 **	0.44 **	0.37 **	−0.18 **	0.64 **	−0.19 **	−0.20 **	0.24 **
13. Self-worth													–	0.47 **	0.46 **	0.50 **	0.48 **	−0.28 **	0.68 **	−0.33 **	−0.26 **	0.30 **
14. Meaning														–	0.51 **	0.57 **	0.44 **	−0.32 **	0.63 **	−0.24 **	−0.29 **	0.17 **
15. Optimism															–	0.61 **	0.54 **	−0.30 **	0.72 **	−0.28 **	−0.26 **	0.24 **
16. Life satisfac.																–	0.69 **	−0.38 **	0.74 **	−0.31 **	−0.39 **	0.23 **
17. Pos. feelings																	–	−0.36 **	0.71 **	−0.30 **	−0.37 **	0.27 **
18. Neg. feelings																		–	−0.41 **	0.35 **	0.46 **	−0.16 **
19. BIT																			–	−0.37 **	−0.40 **	0.35 **
20. Internalizing																				–	0.51 **	−0.29 **
21. Externalizing																					–	−0.12 *
22. Prosocial																						–

* *p* < 0.05; ** *p* < 0.001.

**Table 4:** Independent *t*-test results for externalizing problems.

							95% CI	
Variable		*M*	*SD*	*t*	*p*	*d*	Lower	Upper
Support	*at–risk* group	11.05	2.66	−2.50	0.013	−0.29	−0.51	−0.06
	*typical* group	11.81	2.64					
Community	*at–risk* group	10.83	2.50	−2.56	0.011	−0.29	−0.52	−0.07
	*typical* group	11.57	2.56					
Trust	*at–risk* group	8.02	2.50	−3.37	<0.001	−0.38	−0.61	−0.16
	*typical* group	9.00	2.57					
Respect	*at–risk* group	9.12	2.31	−4.12	<0.001	−0.47	−0.69	−0.24
	*typical* group	10.34	2.66					
Loneliness	*at–risk* group	8.65	3.05	3.12	0.002	0.36	0.13	0.58
	*typical* group	7.54	3.15					
Belonging	*at–risk* group	10.64	2.69	−30.18	0.002	−0.36	−0.59	−0.14
	*typical* group	11.58	2.58					
Engagement	*at–risk* group	10.60	2.60	−1.67	0.095	−0.20	−0.41	0.03
	*typical* group	11.06	2.32					
Skills	*at–risk* group	9.54	2.48	−2.67	0.008	−0.30	−0.53	−0.08
	*typical* group	10.29	2.46					
Learning	*at–risk* group	10.15	3.11	−4.03	<0.001	−0.46	−0.68	−0.23
	*typical* group	11.39	2.58					
Lack of control	*at–risk* group	8.41	3.59	4.94	<0.001	0.56	0.34	0.79
	*typical* group	6.68	2.92					
Accomplishment	*at–risk* group	9.68	2.80	−3.06	0.002	−0.35	−0.57	−0.12
	*typical* group	10.58	2.52					
Self-efficacy	*at–risk* group	10.44	2.93	−2.27	0.024	−0.26	−0.48	−0.03
	*typical* group	11.05	2.19					
Self-worth	*at–risk* group	9.10	2.64	−3.62	<0.001	−0.41	−0.64	−0.19
	*typical* group	10.09	2.34					
Meaning	*at–risk* group	9.71	2.58	−2.22	0.027	−0.25	−0.48	−0.03
	*typical* group	10.39	2.70					
Optimism	*at–risk* group	9.83	3.01	−4.29	<0.001	−0.49	−0.71	−0.26
	*typical* group	11.09	2.45					
Life satisfaction	*at–risk* group	9.41	2.81	−3.74	<0.001	−0.43	−0.65	−0.20
	*typical* group	10.56	2.68					
Positive feelings	*at–risk* group	9.31	3.22	−3.85	<0.001	−0.44	−0.66	−0.21
	*typical* group	10.59	2.85					
Negative feelings	*at–risk* group	10.26	3.18	5.38	<0.001	0.62	0.39	0.84
	*typical* group	8.32	3.14					
BIT	*at–risk* group	33.01	6.63	−4.48	<0.001	−0.51	−0.74	−0.28
	*typical* group	36.39	6.61					

*Note*. *At–risk* group (*N* = 98 = externalizing levels > 11); *typical* group (*N* = 361); *d* [95% CI] = Cohen’s *d* effect size and interpretation = 0.00–0.19 = *negligible*, 0.20–0.49 = *small*, 0.50–0.79 = *medium*, and 0.80+ = *large.*

**Table 5:** Independent *t*-test results for internalizing problems.

							95% CI	
Variable		*M*	*SD*	*t*	*p*	*d*	Lower	Upper
Support	*at–risk* group	10.77	2.76	−4.94	<0.001	−0.50	−0.69	−0.30
	*typical* group	12.05	2.52					
Community	*at–risk* group	11.10	2.58	−1.77	0.078	−0.18	−0.37	0.02
	*typical* group	11.56	2.55					
Trust	*at–risk* group	8.12	2.65	−3.84	<0.001	−0.39	−0.58	−0.19
	*typical* group	9.11	2.50					
Respect	*at–risk* group	9.12	2.51	−5.50	<0.001	−0.55	−0.75	−0.35
	*typical* group	10.53	2.58					
Loneliness	*at–risk* group	9.48	2.88	8.46	<0.001	0.85	0.64	1.05
	*typical* group	6.98	2.97					
Belonging	*at–risk* group	10.86	2.77	−2.96	0.003	−0.30	−0.49	−0.10
	*typical* group	11.63	2.53					
Engagement	*at–risk* group	10.67	2.48	−1.77	0.078	−0.19	−0.37	0.02
	*typical* group	11.09	2.33					
Skills	*at–risk* group	9.47	2.40	−3.99	<0.001	−0.40	−0.60	−0.20
	*typical* group	10.44	2.46					
Learning	*at–risk* group	10.57	2.85	−3.01	0.003	−0.30	−0.50	−0.10
	*typical* group	11.39	2.66					
Lack of control	*at–risk* group	8.55	3.01	7.40	<0.001	0.74	0.54	0.94
	*typical* group	6.35	2.97					
Accomplishment	*at–risk* group	9.55	2.73	−4.81	<0.001	−0.48	−0.68	−0.28
	*typical* group	10.78	2.45					
Self-efficacy	*at–risk* group	10.43	2.45	−3.03	0.003	−0.30	−0.50	−0.11
	*typical* group	11.15	2.31					
Self-worth	*at–risk* group	9.23	2.41	−4.00	<0.001	−0.40	−0.60	−0.20
	*typical* group	10.19	2.40					
Meaning	*at–risk* group	9.41	2.71	−4.65	<0.001	−0.47	−0.66	−0.27
	*typical* group	10.64	2.59					
Optimism	*at–risk* group	10.03	2.88	−4.46	<0.001	−0.45	−0.65	−0.25
	*typical* group	11.19	2.43					
Life satisfaction	*at–risk* group	8.86	2.61	−8.30	<0.001	−0.83	−1.03	−0.63
	*typical* group	10.99	2.54					
Positive feelings	*at–risk* group	8.95	3.14	−7.06	<0.001	−0.71	−0.91	−0.51
	*typical* group	10.96	2.67					
Negative feelings	*at–risk* group	10.35	3.03	7.73	<0.001	0.77	0.57	0.98
	*typical* group	7.98	3.06					
BIT	*at–risk* group	32.51	6.58	−7.20	<0.001	−0.72	−0.92	−0.52
	*typical* group	37.13	6.32					

*Note*. *At–risk* group (*N* = 146 = internalizing levels ≥ 9); *typical* group (*N* = 313); *d* [95% CI] = Cohen’s *d* effect size and interpretation = 0.00–0.19 = *negligible*, 0.20–0.49 = *small*, 0.50–0.79 = *medium*, and 0.80+ = *large.*

## Data Availability

Data is available upon reasonable request from the corresponding author.
